# Easy-to-Use Guidelines on Protein Intake and Physical Activity Recommendations Derived from the COGFRAIL Study and the Toulouse Frailty Clinic

**DOI:** 10.3390/nu17081294

**Published:** 2025-04-08

**Authors:** Eva Peyrusqué, Gabor Abellan van Kan, Patricia Alvarez Rodriguez, Nicolas Martinez-Velilla, Gaelle Soriano, Marion Baziard, Emmanuel Gonzalez-Bautista, Sandrine Sourdet

**Affiliations:** 1 IHU HealtAge, Gérontopôle of Toulouse, Toulouse University Hospital (CHU Toulouse), Hôpital La Grave, Place Lange, 31059 Toulouse, France; peyrusque.e@chu-toulouse.fr (E.P.); soriano.g@chu-toulouse.fr (G.S.); baziard.m@chu-toulouse.fr (M.B.); emmanuel.scout@gmail.com (E.G.-B.); sourdet.s@chu-toulouse.fr (S.S.); 2 CERPOP (Centre d’Epidémiologie et de Recherche en Santé des Populations), Paul Sabatier University, INSERM UMR 1295, 31000 Toulouse, France; 3 Navarre Institute for Health Research (IdiSNA), Public University of Navarre (UPNA), 31006 Pamplona, Navarre, Spain; patricia.alvarez.rodriguez@navarra.es (P.A.R.); nicolas.martinez.velilla@navarra.es (N.M.-V.); 4 Hospital Universitario de Navarra (HUN)-Navarrabiomed, 31006 Pamplona, Navarre, Spain

**Keywords:** malnutrition, frailty, sarcopenia, older adults, nutritional intervention, physical activity intervention

## Abstract

Background/Objectives: In primary care, tailored physical activity and nutritional counselling are scarce for older adults. Several challenges contribute to this issue, the primary obstacle being limited access to expert healthcare providers. The purpose of this study was to propose a quick, easy-to-implement case-finding tool offering straightforward nutritional and physical activity counselling to overcome these barriers. Methods: Cross-sectional, baseline analysis was performed on 277 participants of the Cognitive Function and Amyloid Marker in Frail Older Adults (COGFRAIL) study, aged 70 years and older with mild cognitive impairment (mini-mental state examination score ≥ 20) and autonomy in daily living activities (ADL ≥ 4). Body composition was assessed using dual-energy X-ray absorptiometry, physical function was assessed using the short physical performance battery (SPPB), and nutrition was assessed using the mini nutritional assessment (MNA). A structured dietary interview was conducted to collect data on a typical daily intake pattern. A second database of 725 autonomous frail older adults from the Frailty clinic was used to test the robustness of the findings. Results: Participants with MNA scores < 24/30 and SPPB scores < 6/12 presented a high percentage of protein (74.1%) and caloric (66.7%) deficiency compared to the other categories. Based on standard daily protein and caloric recommendations, age, and weight, this category had a daily protein-caloric deficit of −19.4 ± 22.7 g and −225.5 ± 430.1 Kcal. Conclusions: Based on the data, an easy-to-use algorithm using MNA and SPPB scores is suggested. This algorithm could serve as an effective tool for guiding nutritional and physical activity counselling for community-dwelling older adults.

## 1. Introduction

Sarcopenia is characterized by progressive, age-related loss of muscle mass, strength, and function. This condition significantly affects global health, mobility disability, quality of life, and overall survival in older adults [1]. In the absence of a pharmacological approach, physical activity, particularly resistance straining, and nutritional intervention, especially adequate protein intake, remain the main strategies for managing sarcopenia [2].

Despite the importance of these interventions, implementation faces significant challenges. These include inadequate access to nutritional counselling, lack of motivation to engage in physical activity programs, insufficient promotion of primary prevention by healthcare providers, and limited knowledge of the best clinical practices for promoting physical activity or the necessary quantity of daily protein intake. Furthermore, the complexity and the cost of the assessments along with a lack of individualized approaches often limit the widespread use of these strategies [3].

Sedentarism and poor nutritional status are consistently associated with sarcopenia [4,5], highlighting the need for targeted interventions, such as nutritional counselling and tailored specific physical activity programs to reduce the incidence and impact of sarcopenia in this population.

Physical activity plays a central role in improving muscle function, yet most recommendations made for older adults focus on suboptimal aerobic exercises (e.g., low-intensity walking or biking activities), particularly when promoting leisure time activities (LTAs). These activities only provide limited cardiovascular effects or improvements in fitness [6]. This is why the American College of Sports Medicine and the American Heart Association recommend at least 7.5 MET-hours/week (i.e., more than 150 min/week) of moderate-intensity activity for substantial health benefits [7], with further benefits seen when physical activity increases to up to 420 min/week (1 h/day) of moderate-intensity activity (21 MET-hours/week) [6,8]. A linear dose-response decreased risk was observed for overall cardiovascular disease, coronary heart disease, stroke, and atrial fibrillation [8]. An extensive guideline was recently published on the benefits of physical activity on healthy longevity [9]. A primary drawback is the idea that occupational physical activity (OPA) could confer cardiovascular health benefits [10]. Evidence from epidemiological studies suggests that these activities (gardening, laundering, different handworks) do not influence or could even increase the risk for cardiovascular disease [8,11]. Finally, health providers rarely recommend resistance training for older adults, neglecting the benefits of this type of physical activity on aging muscle. Resistance training can be as beneficial for older adults as it is for younger participants [12], improving muscle mass and strength and minimizing functional decline. An individualized program with adequate instructions and techniques is safe for older adults [13]. A 2019 position paper recommended a resistance exercise program of 1–3 sets of 1–2 exercises per major muscle group, with 5–8 repetitions at 50–80% of one repetition maximum (1RM), 2 or 3 times per week, incorporating the principles of periodization and progression [13,14].

Regarding nutritional counselling, community-dwelling older adults often lack access to qualified nutritionists or dietitians [3]. With a typical decrease in food intake and diminished intestinal absorption of nutrients, older adults are at an increased risk of protein deficiency, which exacerbates sarcopenia [5]. Although the exact protein intake is still under debate, the majority of studies advocate for higher protein intake recommendations, ranging from 1.0 to 1.2 g/kg/day of protein for healthy older adults, and up to 1.5 g/kg/day for those at risk of malnourishment due to the presence of comorbidities [5,15]. These specific needs of older adults have been overlooked in the clinical practice leading to a deficit in caloric and protein daily intake.

In light of these challenges, this study aims to propose a simple, quick-to-implement case-finding assessment tool with a straightforward intervention in nutritional and physical activity counselling, to overcome some of the exposed barriers. The article also aims to increase awareness of the importance of primary prevention to maintain optimal physical functioning and correct nutritional status.

## 2. Materials and Methods

### 2.1. Study Population

Baseline data from the Cognitive Function and Amyloid Marker in Frail Older Adults (COGFRAIL) study have been used to perform this cross-sectional analysis. COGFRAIL procedures and databases have been described elsewhere [16]. Briefly, COGFRAIL is a single-center observational prospective study with a clinical cohort of 321 older adults. The volunteers were invited to participate in COGFRAIL between January 2017 and February 2020 during their routine attendance at the Frailty Clinic or Memory Clinic at Gérontopôle, Toulouse University Hospital [17]. Participants were included if they were frail, aged over 70 years, presented at most a mild cognitive impairment (mini-mental state examination (MMSE) score ≥ 20) [17], and were autonomous for activities of daily living (ADL ≥ 4) [18]. COGFRAIL participants were excluded from the present analysis if missing values were observed in the mini nutritional assessment (MNA) [19], in the short physical performance battery (SPPB) [20], or when the dietary questionnaire was considered non-reliable. All participants provided written informed consent, and ethical approval was obtained from the institutional research committee (CPP SOOM II) on 2 December 2016 (Registration Number: RC31/16/8753). The protocol was registered at ClinicalTrials.gov (NCT03129269).

A supplementary database of patients from the Toulouse Frailty Clinic was used to ascertain the COGFRAIL results. For this analysis, 725 frail patients aged ≥70 years were included, and body composition was assessed using dual X-ray absorptiometry. This database has already been used to assess many clinical aspects of older adults, and its methodology has been published [4,21]. Two exclusion criteria were applied to this database (MMSE < 20 and ADL < 4) to homogenize with the COGFRAIL study. Participants with missing data on the MNA and SPPB were also excluded. The Ethical Committee of the study-coordinating center approved the data collection for scientific purposes (RnIPH 2024-13). No formal written informed consent was required, as the data collected were part of daily standard clinical care activities.

### 2.2. Assessment of Body Composition by iDXA

iDXA measurements were used to evaluate appendicular lean mass (ALM) and total fat mass (TFM) in kilograms. ALM is the sum of the non-bone and non-fat mass of the four limbs in kilograms [22]. Based on this measurement, the Foundation for the National Institutes of Health (FNIH) defined low ALM as <19.75 kg for men and <15.02 kg for women [23].

### 2.3. Nutritional Status

Nutritional status was assessed using baseline body weight, change in body weight, body mass index (BMI, kgm^−2^) [24], the MNA questionnaire, and the participants’ dietary macronutrient and micronutrient intake.

The MNA scores range from 0 to 30 points. Patients were classified as well nourished (≥24 points), at risk of malnutrition (17– < 24 points), or malnourished (<17 points). For this study, the population was dichotomized into patients presenting with a poor nutritional status (MNA < 24 points, patients at risk of malnutrition or malnourished) and those with a normal nutritional status (MNA ≥ 24).

The participants’ dietary macronutrient and micronutrient intake was assessed using diet history interviews performed annually, with a standardized enquiry performed by trained geriatric dietitians. Proxies were invited to participate in this interview if present during the visit. Diet history was obtained through a detailed interview (lasting about 45 min) on the subject’s usual dietary intake, including all meals, drinks (including alcohol consumption), and snacks. Based on dietitian’s impressions, non-reliable interviews were considered as missing data. This interview collected data on a typical daily intake pattern, including the amount, frequency, and preparation methods. The resulting data were analyzed using Nutrilog^®^ software (v 2.31, Marans France) to calculate daily nutrient intake. Nutrilog^®^ is a professional nutrition web application that uses the CIQUAL/ANSES French Food Composition Database (Agence Nationale de SEcurité Sanitaire de l’alimentation, de l’Environnement et du travail/French National Agency of Health, Safety of Nutrition, Environment, and Employment). The Nutrilog^®^ software allows the conversion of food and beverages into daily energy and protein intake. Daily deficits in caloric and protein intake were obtained by subtracting the reported amount of proteins and calories from theoretical needs as follows:

(a) For protein intake, the ESPEN Expert group recommendation of 1 g of protein intake per kg of weight per day for autonomous older adults was used [5]. Protein deficit was calculated by subtracting the recommended protein intake from the daily intake derived from the nutritional enquiry.
(b) For caloric intake, resting energy expenditure (REE) was calculated using the Harris–Benedict formula [25]. Daily energy expenditure was based on REE multiplied by a coefficient based on the degree of physical activity (assessed using the Saltin–Grimby physical activity level scale) as proposed by the French Nutritional Agency [26,27]. Caloric deficit was calculated by subtracting the recommended caloric intake from the daily intake derived from the nutritional enquiry.

### 2.4. Covariates

Autonomy was assessed using the ADL scale, cognitive status using the MMSE, and obesity using BMI. Handgrip strength was assessed using a handheld Jamar dynamometer (Jamar, Irvington, NY, 215 USA). The best of two attempts with each hand sitting was included [28]. A standardized assessment of gait speed was performed by asking the participants to perform a 4 m walk at their usual pace. Timing began when the command was given, and the time needed to complete the 4 m walk was recorded in seconds [29]. Gait speed (calculated as meters per second, ms^−1^) was used as a continuous variable. The SPPB test was used to categorize the participants into good performers (score of 10–12), medium performers (score 7–9), and bad performers (0–6).

### 2.5. Statistical Analysis

We performed a cross-sectional analysis of the aforementioned registries. All descriptive statistical analyses were performed using STATA v11 (Stata Corp., College Station, TX, USA). Continuous variables were assessed using mean and standard deviation and categorical variables with frequencies and percentages. Considering distribution characteristics of variables, pertinent bivariate analyses were performed in order to establish the existence of statistically significant differences between the study populations. Multivariate regression analyses were performed between MNA/SPPB categories based on taking the best category as a reference to establish the existence of statistically significant differences in protein and caloric intake and deficit, for both ALM and FM. The covariates used to adjust these models were age, sex, MMSE, ADL, and BMI.

## 3. Results

The present analysis included 277 COGFRAIL participants and 726 patients from a Frailty Clinic. Table 1 shows the general characteristics of the two study populations. iDXA parameters were available for 106 COGFRAIL participants (mean age 82.7 ± 5.2; 63.2% women; mean SPPB score 8.4 ± 2.8; mean MNA score 24.9 ± 2.7) and 726 frailty patients (mean age 82.8 ± 5.8; 63.2% women; mean SPPB score 8.2 ± 2.9; mean MNA score 24.6 ± 3.6). We appreciate comparable non-statistically significant clinical parameters between the two populations, with the exception of MMSE due to study characteristics (MMSE score of 24.6 ± 2.9 for COGFRAIL population vs. 26.0 ± 3.0 for frailty patients).

Table 2 shows the COGFRAIL participants categorized by SPPB and MNA. We observed that the patients in the lowest category of SPPB (score of 0–6 points) and MNA (score < 24) presented statically significant worse clinical parameters than those in the other categories after multivariate regression analysis. Indeed, these participants presented lower gait speed (0.5 ± 0.2 m per second vs. 1.0 ± 0.1 m per second) and handgrip strength (15.5 ± 6.6 kg vs. 20.5 ± 7.6 kg), with deficits of nearly 20 g/day for protein intake and 225 kcal/day for caloric intake. The participants with deficits (compared to the French Nutritional Agency recommended dietary intake) also had a statistically significant decrease in ALM of 2 kg (16.1 ± 4.5 kg vs. 18.2 ± 4.2) with an increase of 5 kg in fat mass (29.0 ± 17.9 kg vs. 23.4 ± 6.9) compared to participants in the highest categories of MNA and SPPB. Participants who were good performers (SPPB scores of 10–12) and had a good nutritional status (MNA scores ≥ 24) had a gait speed over 1 ms^−1^ and had no deficit in protein or caloric intake. Intermediate categories also presented intermediate data on protein and caloric intake.

Table 3 shows the clinical parameters of the patients from the frailty clinic. The analysis is consistent with the previous COGFRAIL data, showing that bad performers, also at risk or malnourished, presented greater functional and cognitive declines (gait speed of 0.5 ± 0.1 m per second and an MMSE score of 22.6 ± 2.3) than the other categories based on multivariate regression analysis. A gradient in categories can be observed regarding the number of statistically significant impaired variables.

**Figure 1:**
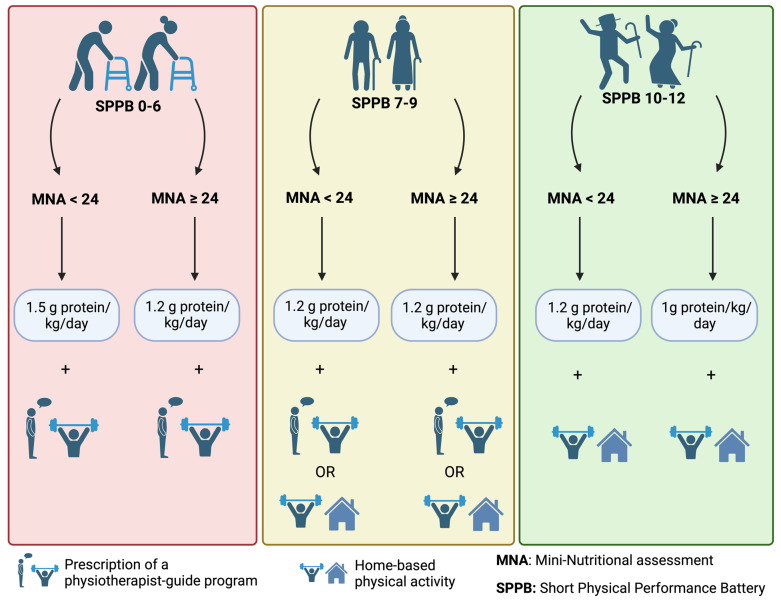
Nutritional and physical activity counselling based on MNA and SPPB. Legend: MNA, mini nutritional assessment; SPPB, short physical performance battery.

Figure 1 shows the potential use of MNA and SPPB scores for the management of older adults. Using the scores of SPPB and MNA (2 well-known geriatric assessment scales), an algorithm could be proposed to guide the management of patients when expert counselling is lacking. Management ranged from a physiotherapist’s prescription or adapted physical activity coaching to nutritional intervention based on different degrees of protein intake.

## 4. Discussion

We propose a person-centered interventional algorithm that provides nutritional and physical activity counselling for community-dwelling older adults, to be used when specific healthcare experts are lacking. Utilizing SPPB and MNA, two well-known geriatric assessment scales, the study population of the two registries could easily be stratified, highlighting differences in physical performance and body composition. Several interventional studies have investigated the use of physical activity and nutritional supplementation to increase muscle strength (dynapenia) and mass (sarcopenia). The results of two systematic reviews indicate that exercise mainly improved muscle strength and physical performance with physical activity, predominantly supervised resistance training. The results were also consistent with the effect of dietary supplementation on muscle mass [30,31]. The novelty, to combine both assessments, is relevant to tailor a personalized nutritional and physical activity intervention.

In our population, the SPPB identified impairments in physical performance. Indeed, SPPB scores of 0–6 identify patients with poor performance, and a supervised resistance training program should be recommended by a physiotherapist. This impaired population will not be able to cope with a self-assessed home-based exercise-program. Participants with SPPB scores of 10–12 were good performers and could participate in a home-based exercise program with or without exercise coaching. Intermediate categories should have a personalized preventive plan, and a home-based program could be helpful with or without a supervised training program, depending on the patient’s characteristics. Safe and effective exercise prescriptions require an assessment of an individual’s health status, baseline fitness, and preferences. For older sedentary patients, an initial prescription of a 30 min daily fast walk could be the first step. Exercise prescription is person-centered and needs to be re-assessed regularly and continuously adapted [32]. Although physiotherapists work on specific muscle domains to improve the observed impairments, the proposed home-based exercises should be a multicomponent (resistance, balance, stretching, and endurance) program. Both programs can be complementary. With this in mind, the European VIVIFRAIL program, which is based on the SPPB score, was designed to address the needs of older adults. This adapted physical activity program includes exercises targeting both the upper and lower limbs, as well as balance exercises [33]. After three months of implementation, the VIVIFRAIL program demonstrated significant improvements in SPPB score, cognitive function, muscle function, and depression [34]. It would be interesting to develop a tool that combines an SPPB-based intervention with one based on the MNA score.

The assessment of MNA identifies patients at risk of malnutrition compared to those with a good nutritional status. Optimal protein intake should be recommended in these participants following current guidelines [5,15,35]. Sarcopenia and impaired body composition parameters are frequently associated with this condition [4]. Expert counselling will still be needed if this first-line intervention is proven to be insufficient, during re-assessment, to ameliorate the global health status of older adults.

Several limitations must be acknowledged. First, the results are Toulouse-based, and external validation is needed using other elderly populations in order to promote the algorithm nationally or internationally. The cross-sectional design also hampers the outcomes. A longitudinal assessment with the specific interventions is needed to determine whether the algorithm can improve functional decline and nutritional impairment. Adherence to interventions by older adults would still be an issue. Nevertheless, the algorithm could promote primary prevention in rural areas where access to specialist counselling is scarce. In these areas, the usefulness of the proposed algorithm must be tested. Although we acknowledge that this tool has to be assessed to prove its validity, its application will improve the management of older adults in primary care.

Finally, the visibility of the proposed algorithm in clinical practice is reinforced by its easy-to-use design, with assessment scales used widely in clinical settings (very feasible in primary care settings). Other key professionals already present in primary care settings (such as nurses, physiotherapists, and other healthcare practitioners) could use this algorithm to enhance their management of older adults. The present study should be expanded in future research by adding new technologies like artificial intelligence or IoT in order to increase its applicability. Interdisciplinary engineering research to develop integrated healthcare-technology projects is in need of simple tools like the proposed algorithm to enhance quality of life of community-dwelling older adults in order to prevent late-life onset of disability [36,37].

## 5. Conclusions

Participants with lower SPPB and MNA scores have lower protein and caloric daily intake. Based on the clinical data of the Toulouse Frailty Clinic and the COGFRAIL study, an easy-to-use algorithm using MNA and SPPB scores could identify community-dwelling older adults who may benefit from nutritional and physical activity counseling. This algorithm needs external validation.

## Figures and Tables

**Table 1:** Clinical characteristics of study populations.

	COGFRAIL (n = 277)	Frailty Clinic (n = 725)	*p* Value
Age, years (mean ± SD)	82.7 ± 5.2	82.8 ± 5.8	0.81
Sex, female (n, %)	175 (63.2)	459 (63.2)	0.96
MMSE (mean ± SD)	24.6 ± 2.9	26.0 ± 3.0	<0.05
ADL (mean ± SD)	5.6 ± 0.5	5.6 ± 0.5	0.98
Gait speed, ms^−1^ (mean ± SD)	0.8 ± 0.2	0.8 ± 0.2	0.59
Handgrip, kg (mean ± SD)	19.1 ± 7.5	20.1 ± 8.4	0.07
BMI (mean ± SD)	26.8 ± 4.9	26.2 ± 5.0	0.10
SPPB (mean ± SD)	8.4 ± 2.8	8.2 ± 2.9	0.18
SPPB 0–6 (n, %)	71 (25.6)	206 (28.4)	0.60
SPPB 7–9 (n, %)	90 (32.5)	237 (32.7)	
SPPB 10–12 (n, %)	116 (41.9)	282 (28.9)	
MNA (mean ± SD)	24.9 ± 2.7	24.6 ± 3.6	0.14
MNA ≥ 24 (n, %)	204 (73.6)	494 (68.04)	0.06
MNA 17- < 24	71 (25.6)	211 (29.06)	
MNA < 17	2 (0.7)	21 (2.89)	
ALM (mean ± SD)	17.7 ± 4.0 (n = 106)	17.4 ± 4.1	0.50
FM (mean ± SD)	24.3 ± 9.0 (n = 106)	24.9 ± 10.2	0.54

Legend: MMSE, mini-mental state examination; ADL, activities of daily living; BMI, body mass index; MNA, mini nutritional assessment; ALM, appendicular lean mass; FM, fat mass.

**Table 2:** COGFRAIL characteristics based on MNA and SPPB (n = 277) and DXA characteristics (n = 106).

N = 277	MNA < 24			MNA ≥ 24		
	SPPB 0–6 n = 27	SPPB 7–9 n = 31	SPPB 10–12 n = 15	SPPB 0–6 n = 44	SPPB 7–9 n = 59	SPPB 10–12 n = 101
Age, years	81.9 ± 6.0	83.5 ± 5.4	82.8 ± 4.2	83.4 ± 5.6	83.7 ± 5.4 *	81.9 ± 4.7
Sex, female (n, %)	21 (12.0)	25 (14.3)	8 (4.6) *	27 (15.4)	39 (22.3) *	55 (31.4)
MMSE, score	22.6 ± 2.3 *	23.5 ± 2.9	26.2 ± 2.9	24.7 ± 2.6	24.9 ± 2.9	24.8 ± 3.0
BMI, Kgm^−2^	27.9 ± 6.8	25.3 ± 5.4	25.2 ± 4.4	27.2 ± 4.3	19.1 ± 7.8 *	26.0 ± 3.8
Handgrip, Kg	15.5 ± 6.6	17.5 ± 6.2	22.1 ± 7.0	18.1 ± 7.6	19.1 ± 7.8	20.5 ± 7.6
Gait speed ms^−1^	0.5 ± 0.2 *	0.8 ± 0.1 *	1.0 ± 0.1	0.6 ± 0.1 *	0.8 ± 0.2 *	1.0 ± 0.1
ALM, Kg	16.1 ± 4.5	14.0 ± 2.6	17. 2 ± 4.4	19.0 ± 3.5	18.3 ± 3.5	18.2 ± 4.2
FM, Kg	29.0 ± 17.9	20.9 ± 8.9 *	19.1 ± 9.1	26.2 ± 8.9	26.9 ± 8.2 *	23.4 ± 6.9
DPI, gr	50.5 ± 16.3 *	58.2 ± 19.3 *	59.2 ± 17.8 *	66.7 ± 15.4	69.8 ± 22.3	70.6 ± 17.9
Deficit proteins, g	−19.4 ± 22.7 *	−3.7 ± 20.0	−4.8 ± 14.3 *	−2.5 ± 19.5	−2.2 ± 20.1	3.3 ± 19.2
DKI, Kcal	1282.0 ± 354.6 *	1414.4 ± 398.9	1506.1 ± 474.4	1571.2 ± 343.9	1578.1 ± 389.5	1610 ± 357.4
Deficit in Kcal	−225.5 ± 430.1 *	−12.18 ± 344.1	−10.6 ± 359.6	53.1 ± 340.1	−10.5 ± 369.5	8.74 ± 376.4

Legend: MNA, mini nutritional assessment; SPPB, short physical performance battery; MMSE, mini-mental state examination; BMI, body mass index; ALM, appendicular lean mass; FM, fat mass; DPI, daily protein intake; DKI, daily Kcal intake. Bold variables * are statistically significant (*p* < 0.05) compared to the variable from the reference category (SPPB 10–12 and MNA ≥ 24) using multivariate regression analyses (adjustments for age, sex, ADL, MMSE, and BMI). Deficit: compared to the French Nutritional Agency recommended dietary intake.

**Table 3:** Frailty Clinic characteristics based on MNA and SPPB (n = 725).

N = 725	MNA < 24			MNA ≥ 24		
	SPPB 0–6 n = 98	SPPB 7–9 n = 71	SPPB 10–12 n = 62	SPPB 0–6 n = 108	SPPB 7–9 n = 166	SPPB 10–12 n = 220
Age, years	84.2 ± 6.0 *	84.3 ± 5.4 *	81.2 ± 5.4	84.2 ± 5.7 *	84.0 ± 4.9 *	80.7 ± 5.8
Sex, female (n, %)	73 (15.9)	43 (9.4)	37 (8.1)	77 (16.8)	98 (21.4)	131 (28.5)
MMSE, score	25.1 ± 3.3	25.1 ± 3 *	26.2 ± 3.0	25.6 ± 3.1	26.3 ± 2.8	26.6 ± 2.9
BMI, Kgm^−2^	24.3 ± 5.2 *	23.5 ± 4.2 *	23.6 ± 5.2 *	29.2 ± 5.1 *	27.2 ± 4.3	26.41 ± 4.3
Handgrip, Kg	15.4 ± 6.9 *	19.3 ± 7.1	22.7 ± 8.9	17.7 ± 7.4 *	20.4 ± 7.9	22.7 ± 8.7
Gait speed ms^−1^	0.5 ± 0.1 *	0.8 ± 0.1 *	1.0 ± 0.1	0.6 ± 0.2 *	0.8 ± 0.2 *	1.0 ± 0.2
ALM, Kg	15.7 ± 3.3	15.7 ± 3.7	16.7 ± 4.4	17.8 ± 3.6	18.0 ± 4.2	18.2 ± 4.3
FM, Kg	21.7 ± 9.4	20.3 ± 9.1	18.7 ± 10.0	31.4 ± 11.2	26.7 ± 8.7	25.1 ± 9.1

Legend: MNA, mini nutritional assessment; SPPB, short physical performance battery; MMSE, mini-mental state examination; BMI, body mass index; ALM, appendicular lean mass; FM, fat mass. Bold variables * are statistically significant (*p* < 0.05) compared to the variable from the reference category (SPPB 10–12 and MNA ≥ 24) using multivariate regression analyses (adjustments for age, sex, ADL, MMSE, BMI, and handgrip-strength).

## Data Availability

The original contributions presented in this study are included in the article. Further inquiries can be directed to the corresponding author(s).
